# The Role of Interfacial Energetics and Defects on The Efficiency of Tin Perovskite Solar Cells

**DOI:** 10.1002/smll.202600068

**Published:** 2026-05-20

**Authors:** Mahmoud H. Aldamasy, Artem Musiienko, Marin Rusu, Davide Regaldo, Zafar Iqbal, Shengnan Zuo, Hannes Hampel, Chiara Frasca, Ahmed G. Bedir, Guixiang Li, Giuseppe Nasti, Meng Li, Jorge Pascual, Qiong Wang, Thomas Unold, Antonio Abate

**Affiliations:** ^1^ Helmholtz‐Zentrum Berlin für Materialien und Energie GmbH Berlin Germany; ^2^ Egyptian Petroleum Research Institute Cairo Egypt; ^3^ IPVF Institut Photovoltaïque d'Ile‐de‐France Palaiseau France; ^4^ Laboratoire de Génie Electrique et Electronique de Paris, Université Paris‐Saclay, CentraleSupé lec CNRS Gif‐sur‐Yvette France; ^5^ Laboratoire de Génie Electrique et Electronique de Paris, Sorbonne Université CNRS Paris France; ^6^ Department of Chemistry Bielefeld University Bielefeld Germany; ^7^ School of Materials Science and Engineering Southeast University, Nanjing Jiangsu China; ^8^ Department of Chemical Materials and Production Engineering University of Naples Federico II Naples Italy; ^9^ Key Lab for Special Functional Materials of Ministry of Education National and Local Joint Engineering Research Center for High‐efficiency Display and Lighting Technology School of Materials Science and Engineering and Collaborative Innovation Center of Nano Functional Materials and Applications Henan University Kaifeng China; ^10^ Instituto de Tecnología Química (ITQ), Consejo Superior de Investigaciones Científicas‐Universitat Politècnica de València Av. dels Tarongers València 46022 Spain

**Keywords:** charge extraction, energy levels of charge selectin contacts, lead‐free perovskite solar cells, tin perovskite solar cells

## Abstract

Tin perovskites are emerging as a sustainable alternative to lead‐based photovoltaics, yet their efficiency remains limited by energy‐level mismatch and intrinsic instability from tin oxidation. Progress is further hindered by inconsistent electrical behavior, obscuring the true bottlenecks of device performance. Here, we deliver a comprehensive, layer‐resolved analysis of FASnI_3_ solar cells, combining Kelvin probe and photoelectron yield spectroscopy to directly map the band structure and quantify interfacial losses. We reveal that the commonly overlooked Bathocuproine buffer layer plays a decisive role in boosting open‐circuit voltage by forming a hybrid energy level with silver, enabling efficient electron extraction.Using time‐resolved surface photovoltage, we uncover the ultrafast charge‐transfer dynamics governing device operation. These insights expose a critical limitation in the conventional p‐p‐n, where severe recombination at the perovskite and hole transport interface suppresses performance. A transition to an n‐p‐p configuration significantly enhances charge extraction and minimizes recombination losses.By integrating these findings into a predictive digital twin, we establish a clear, experimentally validated roadmap toward tin perovskite solar cells exceeding 25% efficiency. This work provides both fundamental understanding and actionable design rules, accelerating the development of high‐performance, lead‐free photovoltaics.

## Graphical abstract

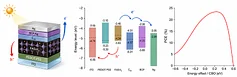

## 1 Introduction

Perovskite materials have demonstrated outstanding performance in various photovoltaic applications due to their optimal charge‐transport properties and tunable bandgap. With a record efficiency of over 26%, perovskite solar cells (PSCs) have matured to the point where they stand on the threshold of commercialization [1, 2]. Nevertheless, these ambitions collide with several obstacles that may delay or, in the worst‐case scenario, prohibit the market reach of PSCs. Among those are lead toxicity and bioavailability [3, 4], which would constitute an imminent danger in the event of cell rupture or cleavage. In this context, tin is the most suitable environmentally friendly lead substitute due to size and electronic similarity [5]. However, the best‐performing tin perovskite solar cells (TPSCs) show efficiencies over 10% lower than their lead counterparts and lag even further behind regarding stability and reproducibility [5, 6]. One of the leading causes of the low efficiency is energy band misalignment, as shown in Figure S1.

Tin perovskites show a shallower valence band maximum (VBM) and conduction band minimum (CBM) than lead perovskites [7]^.^ As a result, the energy bands of the most widely used charge transporting layers (CTLs) are misaligned with the VBM and CBM of the tin perovskite absorber material [7, 8]. This energetic mismatch produces low charge extraction efficiency [9]. Consequently, tin perovskites exhibit lower photovoltaic performance than lead perovskites and have a theoretical efficiency of up to 31% [10, 11]. Practically, the tin perovskite community first adopted the standard *n*‐*i*‐*p* device architecture and then relied on metal oxides such as TiO_2_ and Nb_2_O_5_ as electron‐transporting layers (ETLs) and spiro‐OMeTAD as the hole‐transporting layer (HTL). However, this device architecture suffered from chemical and physical complexities due to the reactivity of metal oxides [12]. With the emergence of fullerene application in PSCs [13], the tin perovskite solar cell device structure was switched to the inverted *p‐i‐n* architecture based on poly (3,4‐ethylene dioxythiophene)‐poly (styrene sulfonate) (PEDOT: PSS) as HTL due to its easy processability and good wettability, and C_60_/BCP as ETL. The later structure was globally adopted and matured to the point that it achieved a power conversion efficiency (PCE) of over 15% despite the considerable energy mismatch at both interfaces [6, 14]. Therefore, it is crucial to manage the interfaces energetically to mitigate the interfacial losses [15].

To transform the promising theoretical predictions of TPSCs into realistic devices, an in‐depth electronic study of the TPSCs is needed. For example, we need an accurate energy‐band diagram to quantify the energy‐band offsets at each interface. Additionally, we need to understand and quantify the effects of interfacial energy misalignment and *p*‐doping concentration on charge extraction dynamics and device performance, setting a bar for each parameter to achieve. Moreover, we need to compare the charge‐extraction dynamics and loss mechanisms of tin perovskites with those of lead perovskites to understand the electronic differences between the two systems, which would help determine which strategy is exchangeable. Such detailed studies are required to fill the knowledge gap on the electronics and energetics of tin perovskites. However, to the best of our knowledge, there are no comprehensive studies of the electronic and charge‐transport properties of tin halide perovskites compared to lead perovskites. It is worth noting that measuring the precise energy‐level positions and interfacial charge‐transfer rates in TPSCs is challenging, especially in thin‐film stacks. The vacuum conditions and energetic excitation beams could harm sensitive tin perovskite films during measurements, underscoring the need to develop suitable characterization techniques tailored to the unique properties of tin perovskites.

In this work, we use non‐destructive techniques to study the photovoltaic, optoelectronic, and charge‐transport properties of tin perovskites through a layer‐by‐layer investigation of the most prevalent tin perovskite device stack. We used an ambient‐pressure Kelvin probe method (KP) and photoelectron yield spectroscopy (PYS) under an inert N_2_ atmosphere to construct the band diagram for tin perovskite devices and quantify the energetic mismatch at each interface, and to estimate the contribution of each layer to the overall open‐circuit voltage (*V*_oc_)of the device [16]. Then, we accurately measured the *p*‐doping concentration in the prepared FASnI_3_ films using the Hall effect, photoluminescence spectroscopy (PL), and KP‐PYS. Furthermore, we used time‐resolved surface photovoltage (trSPV) to study charge extraction rates and to develop a comprehensive understanding of the interfacial charge‐extraction dynamics of different HTLs and ETLs, including self‐assembled monolayers (SAMs) [17, 18]. Furthermore, we quantitatively analyze charge extraction efficiency and the mechanisms responsible for charge loss by conducting charge extraction and recombination simulations for tin and lead perovskites to understand the performance‐limiting factors and the primary charge loss mechanisms in tin perovskites. Finally, we simulate the performance of tin perovskite devices as a function of *p*‐doping concentration and conduction band offset (CBO). Then, we identify the optimum value of each parameter to achieve the highest possible PCE. Our results introduce a comprehensive understanding of the energetic landscape in tin perovskites and provide a road map toward highly efficient tin perovskites with a PCE of over 20%.

## 2 Results and Discussion

### 2.1 The Doping Concentration of FASnI_3_

**FIGURE 1:**
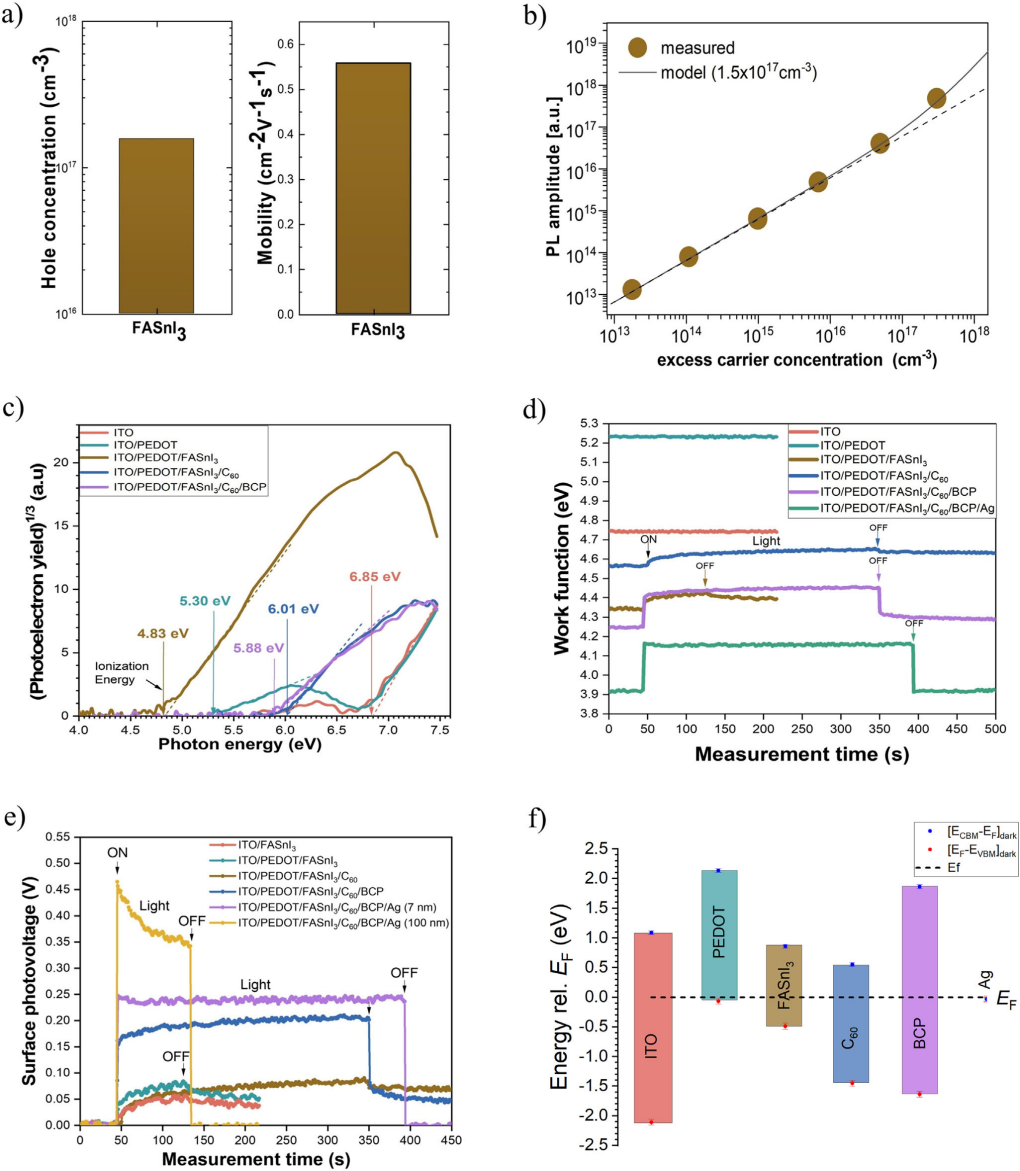
The electronic and energetic landscape of the ITO/PEDOT:PSS/FASnI_3_/C_60_/BCP/Ag solar cell. (a) Charge carrier concentration and mobility of FASnI_3_ thin films. (b) Doping concentration was determined by fitting the PL amplitude as a function of excess carrier concentration from TRPL measurements. (c) Photoelectron yield spectra of each layer of the ITO/PEDOT:PSS/FASnI_3_/C_60_/BCP/Ag device stack with the revealed ionization energies from layer‐by‐layer studies. (d) Time‐dependent WF measurements in the dark and under illumination measured layer‐by‐layer from ITO to the complete solar cell stack. (e) SPV evolution from the ITO film to the full device stack ITO/PEDOT:PSS/FASnI_3_/C_60_/BCP/Ag revealed by KP layer‐by‐layer measurements. (f) The energy band diagram of the ITO/PEDOT:PSS/FASnI_3_/C_60_/BCP/Ag structure with respect to the Fermi level (*E*_F_) is plotted based on the data determined from KP and PYS measurements, the error bars are of 30 meV.

To demonstrate the limitations of TPSCs and to construct a roadmap for further development, careful characterization is needed to determine the doping density, free charge mobility, valence and conduction band energies of the perovskite and selective layers, the bandgap energy, and the bulk and surface recombination rates. Therefore, we applied a set of specialized experimental methods to characterize the layers and interfaces of the most common tin perovskite device stack. Accurate determination of the *p*‐doping level (hole concentration) in FASnI_3_ is highly demanded to understand the electronic behaviour and charge‐carrier dynamics. However, the reported *p*‐doping level of FASnI_3_ is not accurately measured, and the values in the literature range from 10^15^ to 10^18^ cm^−3^ [15, 17]. Therefore, we decided to measure the *p*‐doping in neat FASnI_3_ using the Hall effect, PL, and KP‐PYS techniques. Figure 1a,b shows the hole concentration of the prepared FASnI_3_ with 10% SnF_2_ film. The Hall effect and PL techniques show the same concentration of 1.5 × 10^17^ cm^−3^. In assertive agreement with these values, on freshly prepared FASnI_3_ thin films on quartz glass and indium tin oxide (ITO) substrates, the KP‐PYS measurements reveal hole concentrations of 1.9 × 10^17^ and 1.3 × 10^17^ cm^−3^, respectively. Those values are calculated with the determined E_F_‐E_VBM_ energies of 0.01 and 0.08 eV from KP‐PYS measurements (see next chapter) and hole effective mass in FASnI_3_ of 0.05 *m_0_* as reported by Xie et al. The consistency of the measured hole concentration from Hall effect, PL, and KP‐PYS demonstrates that the 0.05 *m*_0_ hole effective mass, which fits the experimental data from these different measurement methods, is the most accurate value compared to the rest reported in the literature [18].

The doping concentration was determined using three independent methods based on different physical phenomena, namely Hall effect, photoluminescence (PL), and photoemission, providing cross validation of the extracted values. Although Hall measurements can be influenced by ion migration in highly resistive perovskite samples with resistivity above 1 Ohm cm, the resistivity of the films studied here is approximately 100 Ohm cm. Using a representative ion mobility of about 1 × 10^−6^ cm^2^ V^−1^ s^−1^ and the upper reported concentration of mobile ions in this material system, the ionic transport contribution is expected to be several orders of magnitude lower than the free carrier contribution based on a mobility times concentration estimate [19]. Therefore, the Hall derived doping values are considered reliable for the present films [20, 21].

### 2.2 Energy Band Diagram of the ITO/PEDOT:PSS/FASnI_3_/C_60_/BCP/Ag Solar Cell

To understand the efficiency losses related to CTLs in TPSCs, we analyzed the interfacial energy bands alignment between the absorber layer and different CTLs in addition to the charge carrier extraction dynamics. The determined positions of the conduction and valence bands of different tin perovskite compositions and CTLs scatter significantly in the literature. This is partly explained by the difficulty of getting consistent measurements due to challenges like Sn^2+^ sensitivity to oxygen, beam damage during high‐energy photoelectron analysis, and composition changes under high vacuum conditions. Therefore, we performed the KP‐PYS measurements under an inert N_2_ atmosphere under normal conditions and employed low‐energy photons in the 3.4–7.5 eV range. Based on the aforementioned measurements, we built the band diagram for the studied ITO/PEDOT:PSS/FASnI_3_/C_60_/BCP/Ag solar cell device. The Fermi level position is found from work function (WF) measurements by KP (Figure 1d), and the band bending and the contribution of each layer to the solar cell *V*_OC_ are found from the magnitude of the SPV signal derived from KP data in the dark and under illumination (Figure 1e). The VBM position is determined as the ionization energies found from PYS spectra, which were found by linear regression and extrapolation of the Y^1/3^(ℎν) curves in Figure 1c and Figures S2–S5, except that of Ag, which was determined separately from Y^1/2^(ℎν) plots valid for measurements on metals. Knowing the VBM position, the CBM position is found by adding the bandgaps of the respective thin films to the *E*_VBM_. Assuming flat band conditions under the used 100 mW/cm^2^ illumination, the doping concentration of the films is calculated. It is worth mentioning that the PYS measurements for the determination of the ionization energy (*E*_i_) and the KP measurements for the determination of the WF, which determine the Fermi level (*E*_F_) position with respect to the vacuum level, were performed with the same Kelvin tip on the same sample position. Note that the WF of the KP tip was checked each time before starting a new measurement. Furthermore, repeatedly performed PYS measurements have delivered the same ionization energy values denoting the chemical stability of the investigated thin film surface [7, 16, 22, 23].

Figure 1d shows the layer‐by‐layer WF measurements in the dark and under illumination of the TPSC device. The measurements were stable and reproducible over time, proving the prepared films’ surface chemical and electronic stability. The light‐induced WF variations in Figure 1d and the corresponding SPV development in Figure 1e were first observed after the deposition of the FASnI_3_ film. Since no SPV was recorded from ITO and PEDOT:PSS films, the observed SPV amplitude of about 70 mV is attributed to the potential barrier at the FASnI_3_ film surface due to band bending. The C_60_ layer, although deposited as an ETL on top of the FASnI_3_ absorber film, did not enhance the SPV amplitude. Note that the SPV measured from a single film would be attributed to the surface band bending, while an SPV measured from a *p‐n* junction shall coincide with the solar cell device's *V*_OC_. With well‐passivated interfaces and appropriate contacts, the *V*_OC_ of a photovoltaic device is expected to approach the internal voltage (related to quasi‐Fermi level separation) within the absorber, as indicated in Figure 1e (yellow curve), in which the SPV signal of the full device stack matches the *V*_OC_ value obtained by the current density–voltage (*J*–*V*) curve [24, 25] (Figure S6). Therefore, we conclude that the C_60_ layer has no significant contribution to the built‐in voltage within the FASnI_3_ absorber. The SPV of the device stack increases significantly only after the deposition of the next BCP film. A considerable increase in the SPV signal is observed after depositing a semi‐transparent 7 nm‐thick Ag layer. Since the sample is illuminated through the Ag film, the intensity of the incident light is significantly decreased. Thus, the recorded SPV deviates from the potentially maximum achievable SPV signal. Nevertheless, these results show that the internal voltage within the FASnI_3_ absorber increases essentially after the BCP deposition while reaching its maximum after the completion of the device structure with the Ag film. In Figure 1e, we observe a sharp increase of the SPV signal to its maximum value by switching on the light and a sharp decrease of the SPV after switching off the illumination only after completing the solar cell stack with the Ag film. This shows that an optimum charge separation occurs at the back contact after the Ag film deposition. Although the SPV curve for the BCP completed device demonstrates an improved transfer of electrons from the absorber film to the BCP surface, the long SPV relaxation time after the illumination is switched off denotes the presence of surface trap states.

Using data from KP and PYS measurements, we plotted the complete band diagram of the ITO/PEDOT:PSS/FASnI_3_/C_60_/BCP/Ag device stack under study in Figure 1f. We aligned it to the Fermi level for discussion of current transport and to the vacuum level, as shown in Figure S7, to collect data on the electron affinity (EA), Fermi level position, and ionization energy for all layers in the device stack. The Fermi‐level‐aligned band diagram reveals the energy‐level offsets at the perovskite HTL/CBM and VBM/ETL interfaces. Those band offsets influence both charge‐carrier extraction and recombination. For the PEDOT:PSS/FASnI_3_ interface, we calculated a positive band offset Δ*E*_v_ = 0.42 eV by using Δ*E*_v_ = (*E*_F_^pero^ – *E*_i_^pero^) – (*E*_F_^HTL^ – *E*_i_^HTL^). Thus, under the assumption of a low density of recombination states at this interface, an effective extraction of holes from the FASnI_3_ absorber would be expected. However, the extraction of holes into the bulk of the degenerated ITO film will be hindered by its superficial layer, which appears intrinsic, as depicted in Figure 1f. At the FASnI_3_/C_60_ interface, we calculate a negative band offset Δ*E*_c_ = ‐0.30 eV by using Δ*E*_c_ = (EA^ETL^ – *E*_F_^ETL^) – (EA^pero^ – *E*_F_^pero^). Consequently, no barriers exist for an effective electron extraction at this interface. However, since the SPV signal differed slightly from that of the FASnI_3_ film and increased slowly under illumination while showing a long decay tail after the light switch‐off, we assume a high density of trap states at this interface. The band structure of the BCP, as aligned to the C_60_ energy levels, suggests a large barrier for the extraction of electrons. Given the significant increase in the SPV signal upon adding the BCP layer, one can assume that gap states assist electronic transport through the BCP film. However, our KP‐PYS investigations show that the BCP film's chemistry and electronics are strongly influenced by the deposition of the Ag layer, which we will discuss further in the next chapter.

### 2.3 The Electronic Structure of the BCP/Ag Interface

**FIGURE 2:**
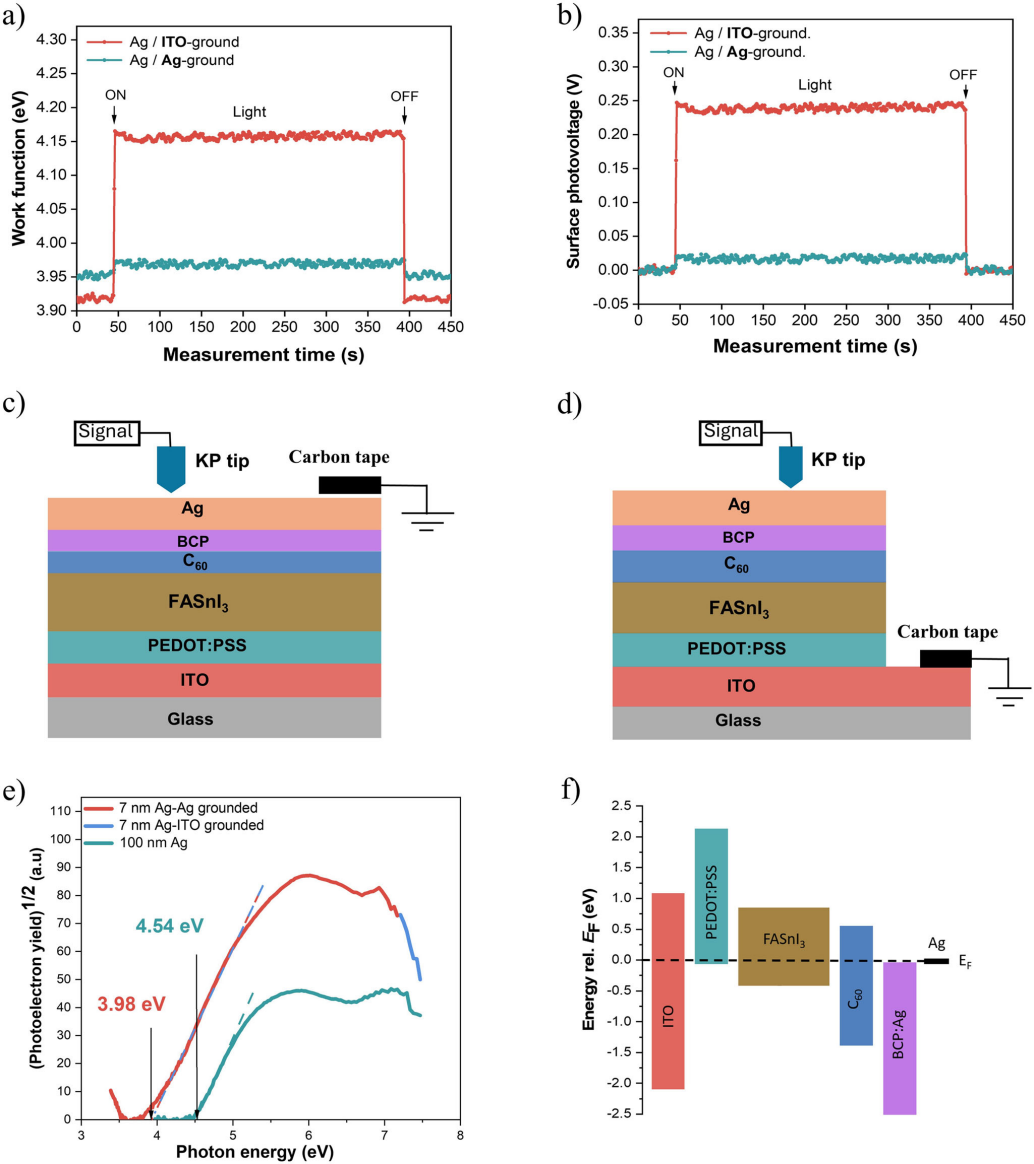
Electronics of the Ag/BCP interface. (a) WF and (b) surface photovoltage of the ITO/PEDOT:PSS/FASnI_3_/C_60_/BCP/Ag device from KP measurements by selectively grounding either the ITO or 7 nm Ag films. (c) Schematic diagram of the KP‐PYS Ag‐grounded sample. (d) Schematic diagram of the KP‐PYS ITO‐grounded sample. (e) Photoelectron yield spectra of a 7‐nm‐thick Ag film on the BCP layer were measured by grounding the device stack to either the ITO or the Ag film. (f) Proposed energy band diagram with a BCP:Ag blend layer with a LUMO level of 4.0 eV, the error bars are of 30 meV.

The BCP layer was inserted as a buffer between the ETL and the cathode to create a cascade of energy levels for improved electron extraction and to simultaneously create a wide hole‐injection barrier that reflects holes, thus preventing interface recombination [26]. However, the remarkable enhancement of the device SPV caused by the BCP/Ag stack motivated us to investigate the BCP/Ag interface to understand the origin of such an enhancement from an electronic point of view. Therefore, we prepared an entire device stack with a top Ag film of only 7 nm thickness. We carried out the KP measurements in the dark and under illumination by grounding the ITO film in the first measurement, while in the second measurement, we grounded the Ag film, as shown in Figure 2a–d. The KP‐PYS measurements were performed on the same sample position in both cases. Note that in the case of grounded ITO, the signals were recorded relative to the *E*_F_ of the ITO thin film, while in the case of grounded Ag, the signals were recorded with respect to the *E*_F_ of the Ag layer. From the dark KP measurements (Figure 2a), we observe almost identical WF values between 3.92 and 3.95 eV for both grounding conditions. Meanwhile, from the PYS measurement (Figure 2e), we identify an *E*_i_ value of 3.98 eV in both cases. These results show that the WF of an investigated thin film on a solar cell stack is properly determined by KP measurements, independent of whether the measurement is conducted by grounding the solar cell's ITO (bottom) contact or directly on the measured top layer. The SPV of the Ag‐grounded measurement (Figure 2b) is attributed to the (minor) surface band bending of the top layer. In contrast, the SPV of the ITO‐grounded plot is attributed to the *V*_OC_ of the stack, which is related to the Fermi level splitting due to the generation of charge carriers under illumination in the FASnI_3_ absorber thin film. Thus, both KP (dark) and PYS measurements reveal similar WF and *E*_i_ values, respectively, as expected for a measurement on a metal or a degenerated semiconductor. However, the measured value of about 4.0 eV is (i) 0.56 eV lower than that of the 100 nm thick Ag reference film (Figure 2e), (ii) 0.25 eV lower than the *E*_F_ of BCP, and (iii) 1.88 eV lower than the *E*_i‐BCP_. Therefore, we suggest that the early deposited Ag diffuses into the BCP layer, forming a BCP:Ag blend with a WF of around 4 eV, which is in an optimum position for effective electron extraction from C_60_ [27, 28].

The Ag:BCP complex was introduced by Ying et al. as a mediator between C_60_ and the indium‐zinc oxide (IZO) transparent electrode to protect C_60_ from damage during sputtering. They demonstrated the formation of Ag:BCP complex [29, 30]. In another context, metal:BCP complexes such as Mg were also reported [31, 32].

On the other hand, our BCP/Ag interface investigation reveals similar energies of about 4.0 eV for the WF and ionization energy. Such findings indicate the formation of a degenerated BCP:Ag surface layer. Therefore, we propose that BCP:Ag is a complex with a Fermi level coinciding with its CBM position at 4.0 eV. This low WF contact layer provides a downward bending of the C_60_ bands, thus enabling a smooth transfer of electrons from C_60_ to the back Ag electrode, as shown in Figure S8. Conclusively, we suggest a modified energy band diagram as indicated in Figure 2f, in which a BCP:Ag blend with a WF ≈ 4 eV replaces the traditional BCP layer [33, 34].

### 2.4 Charge Extraction Dynamics at Charge Selective Contacts

(a) Charges dynamics at the HTL buried interfaces

**FIGURE 3:**
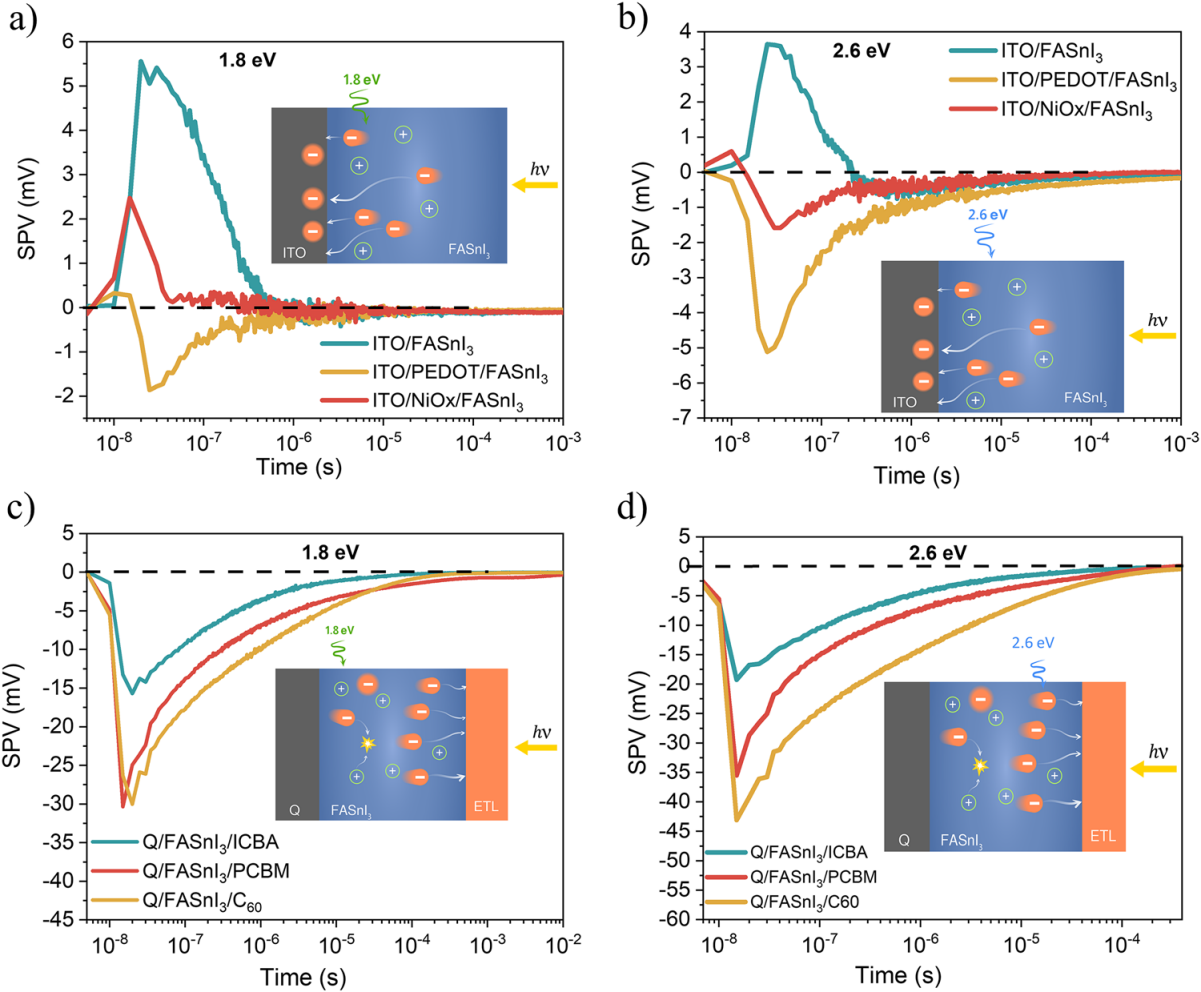
Free electron and hole extraction dynamics in different ETL and HTL interfaces of TPSCs. a,c) excitation using 688 nm laser pulse (1.8 eV), penetrating around 210 nm in a 250 nm‐thick film and exciting electrons near the buried interface. The films were deposited on ITO and quartz (Q) respectively. b,d) Excitation using a 486 nm laser pulse (2.6 eV) penetrating up to 90 nm and exciting electrons in the bulk of the perovskite film.

After illustrating the energetic landscape in the most common TPSC stack, we investigated the ability of different CTLs to extract charges from the absorber layer by measuring trSPV. We illuminated the interfaces under study from the top side, as shown in Figure 3, and recorded charge dynamics from 5 ns to 10 ms. Figure 3a,b insets show the trSPV diagram of neat FASnI_3_ at two different excitation energies, 1.8 and 2.6 eV, corresponding to penetration depths of 210 and 90 nm, respectively [35] as indicated in Figure S9. The sign of the SPV signal reflects the electron or hole separation in space. A positive (negative) sign of transient reflects the electron (holes) separation for the bulk of the thin film to its surface or interface, leaving the perovskite film positively (negatively) charged [36]. The amplitude and rise rate of the signal indicate the number of separated charges and thus express the efficacy of the charge extraction. The subsequent signal decrease indicates the recombination losses of the separated charge carriers. Figure S10.

In the trSPV measurements, the transient response analyzed in this work corresponds to charge carrier dynamics in the range from nanoseconds to hundreds of nanoseconds. The capacitive response of the measurement setup is characterized by a time constant below 1 ms, which is several orders of magnitude slower than the analyzed transient window and therefore has only a minor influence on the fast nanosecond signal. Ionic species in the perovskite are low mobility species with mobilities several orders of magnitude lower than those of free carriers. Therefore, ion migration and ion‐induced field screening are expected to occur on much slower timescales, typically on the order of seconds, rather than in the early nanosecond regime [37]. To further suppress memory effects and charge accumulation, SPV transients were recorded at a low repetition rate of 1 Hz. Ion dynamics in the same material system were previously investigated by our group and were shown to be modulated on the seconds timescale [21].

We start by measuring the ability of ITO to extract charges directly from the FASnI_3_ layer without any CTL. We found that the ITO/FASnI_3_ interface shows a positive trSPV signal, which indicates an electron accumulation near/in ITO, as shown in Figure 3a,b. The peak signal decreased when we generated carriers further away from the interface with 2.6 eV photons (Figure 3b). This electron accumulation can be attributed to the electron trapping or transfer in ITO, as similar effects were observed in ITO/lead perovskite interface previously [36]. Further, we explored hole extraction dynamics in FASnI_3_/HTLs interfaces. In contrast to ITO/FASnI_3_, PEDOT:PSS/FASnI_3_ interface shows negative signals across the perovskite film, revealing hole extraction in PEDOT:PSS. However, the signal is stronger when we generate carriers deeper in the bulk of the perovskite film (Figure 3b) than closer to the interface (Figure 3a). This change in signal amplitude demonstrates a high interfacial recombination rate or barrier at the PEDOT:PSS/FASnI_3_ interface. This draws our attention to the significance of the interfacial passivation and alignment of the buried interface in TPSCs [38, 39]. Notably, PEDOT:PSS shows a small positive signal at the beginning, an early electron trapping across the interface before 10 ns, which is shortly overflooded by hole extraction (Figure 3a). This indicates that the process of charge extraction at the PEDOT:PSS/FASnI_3_ interface is derived by a competition between two processes, hole extraction, and electron trapping, which could be attributed to energy bands misalignment and low charge selectivity of PEDOT:PSS. Nevertheless, the hypothesis on electron trapping is in perfect agreement with data measured at 2.6 eV for the PEDOT:PSS interface (Figure 3b). We did not observe any positive signal due to electron‐hole generation and recombination far from the PEDOT:PSS interface and prevailed majority of free hole drift in the bulk of the *p*‐type material [40].

Next, we studied the hole extraction capabilities of NiOx and two carbazole‐based SAMs (2PACz and MeO‐2PACz, which stand for SAM3 and SAM2, respectively), which previously showed advanced hole extraction capabilities in lead‐based perovskite cells [36] and were also tried for tin perovskites [41]. The trSPV transient of NiOx shows a much less intense negative signal and much worse hole extraction capabilities than PEDOT:PSS. When free carriers are generated closer to the interface (with 1.8 eV photon), electron accumulation at the NiOx/FASnI_3_ interface dominates, giving rise to a positive trSPV signal, as indicated in Figure 3a. At the same time, hole extraction becomes apparent only at 2.6 eV illumination, which is still affected significantly by the positive signal for electron accumulation. The detailed qualitative interpretation of charge transport for the NiOx interface is given in Figure S11. Conversely, only 2PACz showed signs of hole extraction at 2.6 eV illumination among SAMs, as shown in Figure S12. Similar to NiO_x_, SAMs showed electron accumulation and low extraction capabilities compared to PEDOT:PSS. To validate the alignment effect on the hole extraction by SAMs, we added 20% SnBr_2_ to deepen the valence band of tin perovskite [7]. Interestingly, we observe the improvement of hole extraction by NiO_x_, 2PACz, and MeO‐2PACz, which proves such HTLs could work with tin perovskites if the energy bands are appropriately aligned. In conclusion, these HTLs can be optimized for tin‐perovskites with a broader bandgap for lead‐free tandem applications.

(b) Electron extraction dynamics at TPSCs

To explore electron extraction dynamics at perovskite/ETLs interfaces, we measured the trSPV of commonly employed fullerene derivatives indene‐C_60_ bisadduct (ICBA) and [6,6]‐phenyl‐C_61_‐butyric acid methyl ester (PCBM), and C_60_ deposited on top of FASnI_3_ as depicted in Figure 3c,d. In this structure, we illuminate from the top side. Hence, the higher excitation energy (2.6 eV) generates charge carriers close to the interface, while the lower energy laser (1.8 eV) excites near the bulk of the film, as shown in the inset of Figure 3c,d. We observe negative signals for all ETLs, which indicate an active electron extraction at the interface. The rapid increase in the signal amplitude suggests fast separation dynamics of electrons toward the ETL. Generally, C_60_ shows a better extraction, especially when we excite near the interface. Interestingly, the charge extraction rate of the three ETLs follows the same order of charge mobility, which is 1.6, 6.1 × 10^−2^, and 16.9 × 10^−3^ cm^2^ V s^−1^ for C_60_, PCBM, and ICBA, respectively, and not the order of the conduction band offset (CBO) values. This correlation can explain the superior performance of the C_60_‐based devices regardless of the unmatched energy bands compared to ICBA and PCBM, keeping in mind also the vital role of BCP when coupled with C_60_ to form one ETL as explained in a previous section [42]. In addition, the C_60_ interface (as well as other ETLs) shows a much larger peak amplitude of the signal (−47 mV) than the PEDOT:PSS interface (−5 mV), which reveals much better electron extraction capabilities compared to holes. This finding is surprising because electrons, which are minor carriers, are heavily influenced by empty traps in *p*‐type doped FASnI_3_. Therefore, one would expect a lower amplitude of trSPV signals for ETLs interfaces than HTLs interfaces. This observation directly highlights one of the bottlenecks in the state‐of‐the‐art TPSC‐HTL interface.

To investigate electron and hole extraction simultaneously, we compared the trSPV signals for perovskite samples with ETLs and HTLs, as depicted in Figure S13. The results demonstrate a significant enhancement in the extracted charges, with the trSPV signal's amplitude (−160 mV) several times larger than the combined signals of the pure ETL and HTL. This notable increase in the trSPV peak amplitude signifies the strong correlation between electron and hole extraction and highlights the substantial recombination losses caused by unextracted carriers. As carrier recombination occurs through various channels, including non‐radiative, surface, and radiative pathways, Note S1 and Figure S14 provide a more in‐depth discussion.

### 2.5 Simulation of Charge Extraction and Recombination in Tin Perovskite Interfaces

In previous chapters, we qualitatively demonstrated charge transport dynamics in tin perovskite interfaces using various ETLs and HTLs. In this section, we provide a quantitative analysis of charge extraction efficiency and the mechanisms responsible for charge loss by conducting charge extraction and recombination simulations. We simulate charge transport at interfaces that exhibit the highest charge extraction efficiency in tin perovskites and compare the quality of charge extraction with similar interfaces of lead perovskites, (Cs_0.05_FA_0.855_MA_0.095_)Pb(I_0.9_Br_0.1_)_3_ with a power conversion efficiency of 22%. This comparison gives insights into the primary mechanisms causing charge losses and provides valuable information for developing future strategies to enhance tin perovskites and other optoelectronic devices.

**FIGURE 4:**
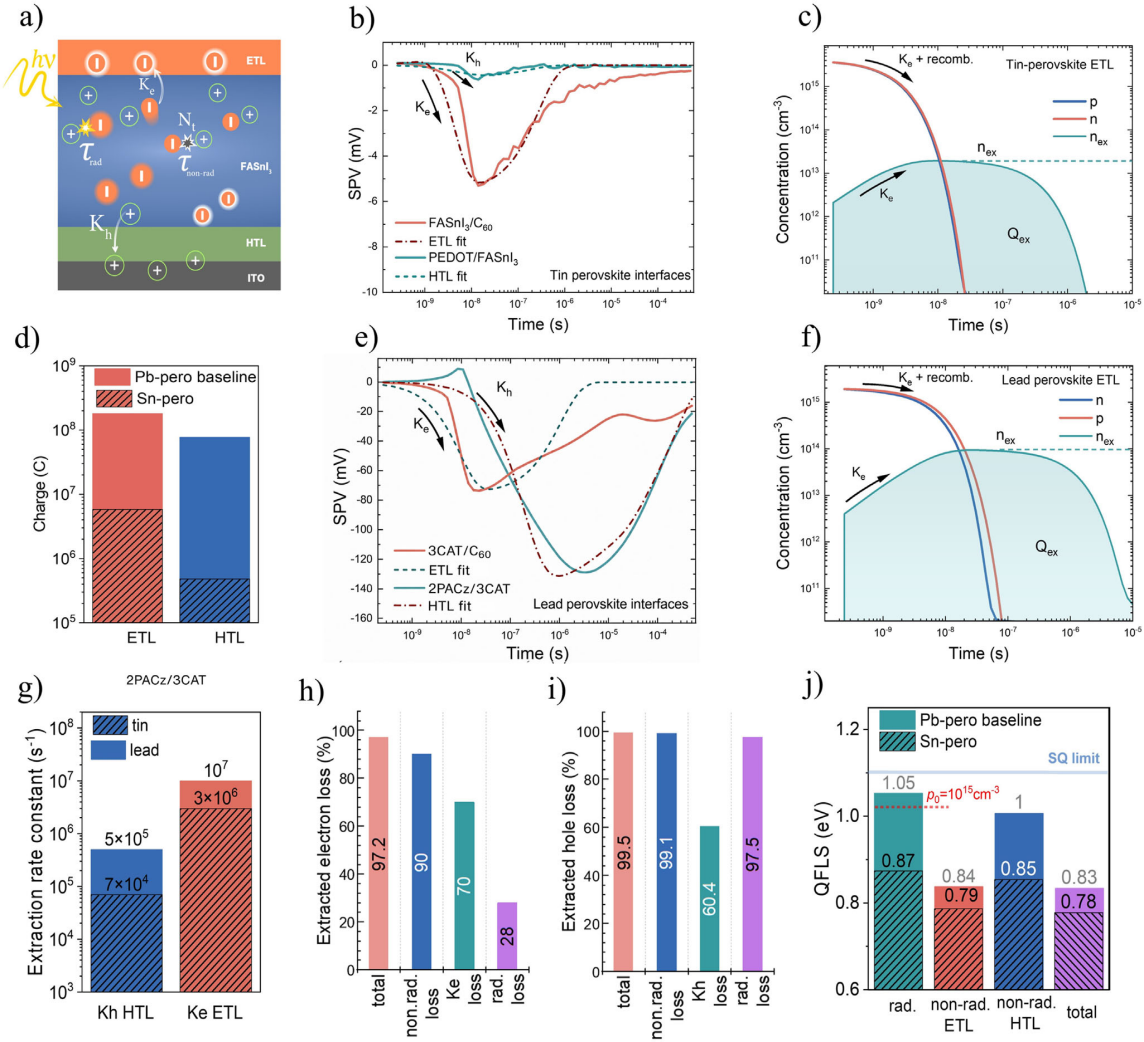
Non‐radiative recombination is the primary charge loss channel in TPSCs according to the simulation of charge extraction and charge losses in FASnI_3_ and (Cs_0.05_FA_0.855_MA_0.095_)Pb(I_0.9_Br_0.1_)_3_ perovskite systems. (a) A theoretical model describing charge extraction. (b) Fitting of experimental data in tin perovskites ETLs and HTLs interfaces. (c) Fitting of experimental data in lead perovskite ETL and HTL interfaces. (d) Total electron and hole charge extracted by HTL and ETL per one laser pulse. (e,f) Time evolution of electron and hole concentrations in the perovskite layer, along with the evolution of extracted electrons in the tin and lead systems, respectively. (g) Hole and electron extraction rate constants. (h,i) Loss of extracted electrons and holes induced by non‐radiative recombination, extraction constant deficit, and radiative recombination in the ETL and HTL, respectively, using the charge in the Pb system as a baseline. (j) Quasi‐Fermi Level Splitting (QFLS) affected by non‐radiative and radiative recombination channels.

There are two channels for charge loss: radiative and non‐radiative recombination. The radiative recombination rate (C_b_∙Δ*n*(*t*)∙(Δ*p*(*t*)+*p*0)) is influenced by the background hole concentration *p*0. The total non‐radiative recombination $\left(\frac{\Delta n}{\tau_{non-rad\_e}}\;and\;\frac{\Delta p}{\tau_{non-rad\_h}}\right)$ consists of bulk $\left(\frac{\Delta n}{\tau_{bulk_{e}}}\right)$, surface ((*s_e_* ∙Δn and *s_h_* ∙Δp), and interface (*s_ie_* ∙Δn and *s_ih_* ∙Δp) contributions, as demonstrated in Equations S1 and S2, where Δn(t) and Δp(t) represent photogenerated electrons and holes, respectively. *s_e_* and *s_h_* are non‐radiative surface recombination rates at the perovskite surface and *s_ie_* and *s_ih_* at the HTL and ETL interface. We consider these recombination channels as key factors governing charge dynamics in perovskite interfaces, along with electron and hole extraction rates *K*e∙*n*(t) and *K*h∙*p*(t), where *K*e and *K*h denote the coefficients for electron and hole extraction rates, respectively, as shown in Equations (1) and (2) and Figure 4a. Further details regarding the model can be found in Equations S1–S4.

(1) $$\frac{d\Delta p}{dt}=-K_{h}\Delta p+K_{hb}\Delta p_{HTM}-C_{b}\left(\left(\Delta p+p0\right)\Delta n\right)-\frac{\Delta p}{\tau_{non-rad\_h}}$$

(2) $$\frac{d\Delta n}{dt}=-K_{e}\Delta n+K_{eb}\Delta n_{HTM}-C_{b}\left(\left(\Delta p+p0\right)\Delta n\right)-\frac{\Delta n}{\tau_{non-rad\_e}}$$

We initially employed the Levenberg‐Marquardt method to fit the experimental data of FASnI_3_/C_60_ and PEDOT:PSS/FASnI_3_, aiming to determine the rate constants that best align with the experimental trSPV results [36, 43]. The fit results are presented in Figure 4b and Table S1, while the time evolution of electrons, holes, and extracted electrons is depicted in Figure 4c. Subsequently, we performed a similar fitting process for the electron and hole extraction dynamics in lead perovskites. We utilized trSPV measurements conducted under identical conditions, as shown in Figure 4e,f and Table S1. Careful examination of the charge extraction dynamics reveals a direct correlation between the rise of the trSPV signal in time (Figure 4b,e) and a reduction in carrier concentration (Figure 4c,f) within the perovskite film. This reduction is a consequence of the charge extraction process, increasing the extracted electron concentration in ETL and HTL. The further decay of trSPV and the maximum amplitude of the trSPV signals are significantly influenced by radiative and non‐radiative recombination processes that tend to diminish the overall carrier density.

The simulation results show significant charge extraction and recombination differences between lead and tin perovskites. The lead perovskite system exhibits lower recombination losses, resulting in a longer carrier lifetime, higher concentration of extracted charges, and an extended exponential tail, as indicated in Figure 4f. Moreover, the lead perovskite demonstrates a larger total extracted charge, Q_ex_, calculated as the surface area under n_ex_(t) and p_ex_(t), as summarized in Figure 4d. Interestingly, the total extracted charge in the lead perovskite system is nearly balanced, with Q_e_/Q_h_ = 2. However, a much more significant difference is observed in the tin perovskite system, with Q_e_/Q_h_ = 14.5. Additionally, the tin perovskite system loses a considerable number of extracted charges. Specifically, the ETL extracts 30 times fewer electrons, and the HTL extracts 160 fewer holes (at the exact condition of 5 ns laser pulse excitation) than the lead perovskite system. This significant reduction in extracted charge highlights the poor capabilities of tin perovskite solar cell interfaces in extracting both holes and electrons, and significant recombination losses.

After demonstrating the humble charge extraction capabilities of tin perovskites compared to lead perovskites interfaces, next, we quantify each recombination channel's impact on the extracted charges. Three factors can influence the extracted charges: the extraction rate constant (rate constant deficit), non‐radiative recombination, and radiative recombination. We considered the charge extracted from lead perovskite as the baseline. We examined the impact of factors affecting charge extraction individually to assess their influence on charge losses, which would reveal a road map to tackle the energy bands misalignment challenge in tin perovskites. We found that tin perovskites exhibit lower charge extraction constants, as illustrated in Figure 4g. The rate constraints for the HTL and ETL are 33 and 12 times lower than those in Pb perovskite interfaces, respectively. This difference may arise from the energetic misalignment between the absorber layer and the CTLs, as highlighted in Figure 1. Alternatively, it could be attributed to the damage on the perovskite surface, resulting in a poorly aligned interface with the CTLs. The charge losses are depicted in Figure 4h,i, where 100% represents the electron and hole charge from Figure 4d, respectively. The charge loss percentage is calculated using the formula loss = (Q_lead_ – Q_tin_)/Q_lead_.

In both ETLs and HTLs, the primary source of charge loss is non‐radiative recombination, accounting for 90% of the charge loss in the ETL interface and 99% in the HTL interface. This slightly higher loss percentage at the HTL interface can be attributed to the larger surface recombination, as indicated in Tables S1 and S2. The extraction rate constant deficit contributes to 70 and 60% of charge loss in the ETL and HTL interfaces, respectively. Interestingly, at the HTL interface, the high material doping (1.5 × 10^17^ cm^−3^) results in significant hole losses, with 97% of holes being lost. In contrast, the same doping level in the ETL induces only 28% of electron loss at its interface. To identify the reasons for such behaviour, we plotted quasi‐Fermi level splitting (QFLS) in both lead and tin systems, demonstrating the effect of recombination channels on charge concentration in the perovskite absorber film itself, considering additional ETL and HTL surface recombination (Figure 4j). Due to the significantly enhanced passivation capability of 2PACz in lead perovskites, QFLS reaches a value of 1 eV (after recalculation of QFLS to the bandgap of FASnI_3_), which is very close to the Shockley‐Queisser limit of 1.1 eV in FASnI_3_. In contrast, the C_60_ ETL interface is more susceptible to non‐radiative recombination, resulting in a QFLS of 0.84 eV. Consequently, doping and radiative recombination substantially influence the charge extraction potential in the HTL interface in tin perovskites compared to the ETL interface.

Several key steps must be taken to achieve the baseline performance of lead perovskite (22% efficiency). First, it is crucial to significantly reduce non‐radiative recombination and passivate trap states both in bulk and on the surface of the absorber tin perovskite material. This is necessary to achieve lifetimes in the microsecond range, similar to those observed in lead perovskites. Secondly, the background hole density needs to be reduced to at least the level of 10^15^ cm^−3^. Thirdly, there is a need to improve the alignment of the ETL and HTL to enhance the extraction rate constants, aiming for values around 10^7^ cm^−3^ s^−1^.

### 2.6 Simulation of Tin Perovskite Solar Cell Operation

We have comprehensively studied the factors influencing energy alignment, charge extraction, and recombination in tin perovskite interfaces. We further investigated the impact of these limiting factors on a representative TPSC device with a PCE of 6%. We used 2D drift‐diffusion simulations under steady‐state illumination conditions to model the device. Based on the findings of KP and PYS measurements, we modelled the perovskite, C_60_, and PEDOT:PSS layers as crystalline semiconductors, considering their respective thicknesses, energy bandgaps, and electron affinities. The perovskite *p*‐type doping was set to 1.5 × 10^17^ cm^−3^, as determined in the present study by the Hall, PL, and KP‐PYS measurements. Additionally, we considered the significant valence band offset (VBO) of approximately 0.53 eV between the perovskite layer and C_60_, as revealed by the KP‐PYS study.

To facilitate the injection of photogenerated holes from the perovskite to PEDOT:PSS via thermionic emission over the barrier, we adjusted the VBM of PEDOT:PSS in our model. We increased the VBM by 0.12 eV compared to our experimental data, thereby modifying E_i‐PEDOT_ (from E_i‐exp_ = 5.30 eV to E_i‐model_ = 5.18 eV), considering the possibility of defect‐assisted tunneling across the barrier. Furthermore, we note that the VBM edge of PEDOT:PSS may shift upon perovskite deposition. Ohmic contacts were implemented at the C_60_ and PEDOT:PSS sides of the solar cell, with corresponding WF values of 3.95 and 4.74 eV, measured for Ag:BCP and ITO, respectively.

**FIGURE 5:**
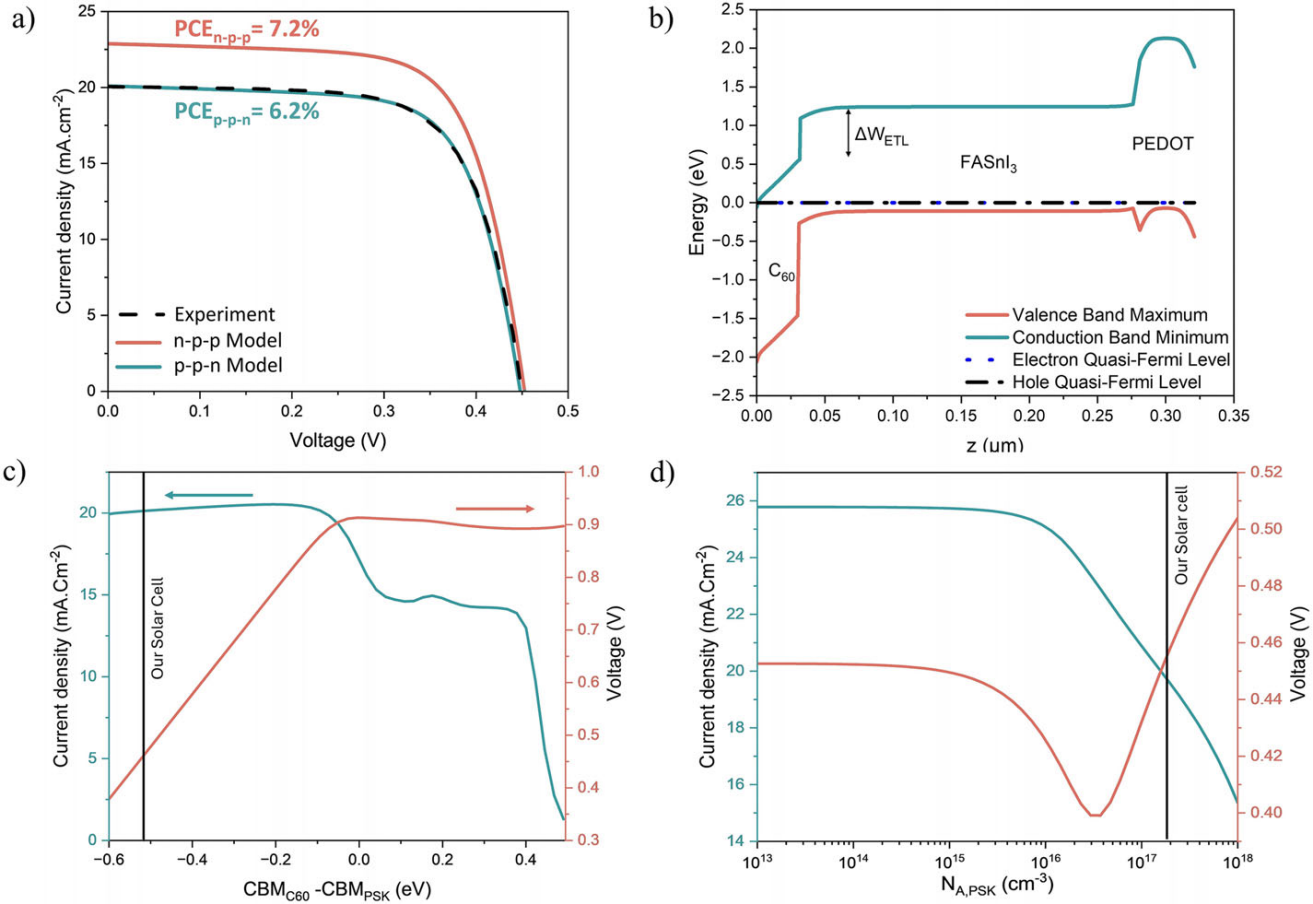
CBO, non‐radiative recombination, and doping limits FASnI_3_ solar cell performance in p‐p‐n and n‐p‐p architecture (a) *J*–*V* curves of the simulated solar cell in *p‐p‐n* and *n‐p‐p* configuration with respect to the experimental p‐i‐n device. (b) The equilibrium band diagram of the solar cell employs the parameters listed in Table S5. (c) *J*_SC_ and *V*_OC_ of the solar cell as a function of the conduction band offset between the perovskite and C_60_, and (d) as a function of the perovskite *p*‐type doping.

Given the considerable CBO of approximately −0.53 eV between the perovskite and C_60_, we anticipated significant non‐radiative recombination at this interface. To address this issue, we introduced a fictitious layer between the two materials with Shockley‐Read‐Hall non‐radiative recombination. This 1 nm‐thick layer shared the CBM of C_60_ and the VBM of the perovskite, enabling recombination between electrons in C_60_ and holes in the perovskite. This additional layer was necessary to align the *V*_OC_ of our simulated perovskite solar cell with the experimental data. We plotted the *J*–*V* curve under 1‐sun illumination of our simulated device (in blue) and the experimental points in Figure 5a. We report all the material parameters employed to produce the blue *J*–*V* curve in Table S5.

Figure 5b illustrates the equilibrium band diagram of the material stack. As mentioned earlier, a significant cliff at the FASnI_3_/C_60_ interface limits the maximum achievable *V*_OC_. Since the perovskite doping density exceeds that of C_60_, most of the built‐in voltage drops across the C_60_ film, resulting in an ineffective separation of photogenerated carriers due to a negligible electric field strength across the perovskite layer. Notably, with a field‐free region, most carriers are generated near the PEDOT:PSS/FASnI_3_ interface. Therefore, electron collection and extraction at the back contact is hindered, considering the low lifetime of the free carriers in FASnI_3_.

Figure 5a also contains the *J*–*V* curve obtained by illuminating the material stack from the C_60_ side (red curve) to test the feasibility of the *n‐p‐p* solar cell architecture. In contrast, all the material parameters are equal to the *n‐p‐p* configuration. If the solar cell was illuminated from the ETL side, most of the electrons would be photogenerated near the C_60_ interface, reducing the impact of bulk non‐radiative recombination on the electrons and constituting the minority carriers in our perovskite layer. Therefore, we found that the n‐p‐p architecture delivers a larger PCE value than the p‐p‐n structure (PCE_n‐p‐p_ = 7.2% compared to PCE_n‐p‐p_ = 6.2%).

After achieving a satisfactory fit for the *J*–*V* curve, we explored variations of energy offset, doping, and recombination rate on the TPSCs efficiency, demonstrating room for further improvement. First, we focused on the CBO at the FASnI_3_/C_60_ interface. Figure 5c shows how TPSCs would perform if we mitigated the large CBO. Starting from the conditions indicated by the solid black vertical line (ΔW_ETL_ = CBM_C60_ – CBM_psk_ = −0.53 eV), *J*_SC_ would not degrade significantly, while *V*_OC_ would nearly double up until CBM_C60_ ≃ CBM_psk_ – 0.1 eV. Increasing the energy gap between CBM_C60_ and VBM_psk_ would reduce non‐radiative recombination between electrons in C_60_ and holes in the perovskite layer, benefiting the *V*_OC_. Importantly, this improvement can be achieved without decreasing the non‐radiative lifetime of the interface layer (or its trap density), which we maintained at 1.5 ns in our simulations. If the CBO increases further, a barrier for electron extraction emerges, severely degrading *J*_SC_. On the contrary, *V*_OC_ would not be affected, as it is determined under zero‐current conditions.

Next, we consider the effect of *p*‐doping density on TPSC *J*_SC_ and *V*_OC_. Starting from the experimental concentration indicated by the solid black vertical line (N_A, pero_ = 1.5 × 10^17^ cm^−3^), reducing the perovskite doping to values below 10^16^ cm^−3^ establishes a constant electric field throughout the perovskite film. This field improves charge extraction, enhances *J*_SC,_ and minimizes bulk non‐radiative recombination. However, *V*_OC_ is slightly affected since it is primarily limited by interface recombination. The non‐monotonous trend of *V*_OC_ around 0.4 V can be attributed to two competing phenomena. On the one hand, increased perovskite doping negatively impacts *V*_OC_ since it further reduces the electric field strength across the perovskite layer. On the other hand, higher perovskite doping values may slightly benefit *V*_OC_ since non‐radiative recombination at the FASnI_3_/C_60_ interface would decrease. As the perovskite doping density increases, the interface Fermi level shifts from values close to the C_60_ CBM toward the perovskite VBM, as illustrated in Figure S15. When the interface Fermi level approaches midgap, the non‐radiative recombination rate at the interface increases since the populations of holes and electrons become comparable.

**FIGURE 6:**
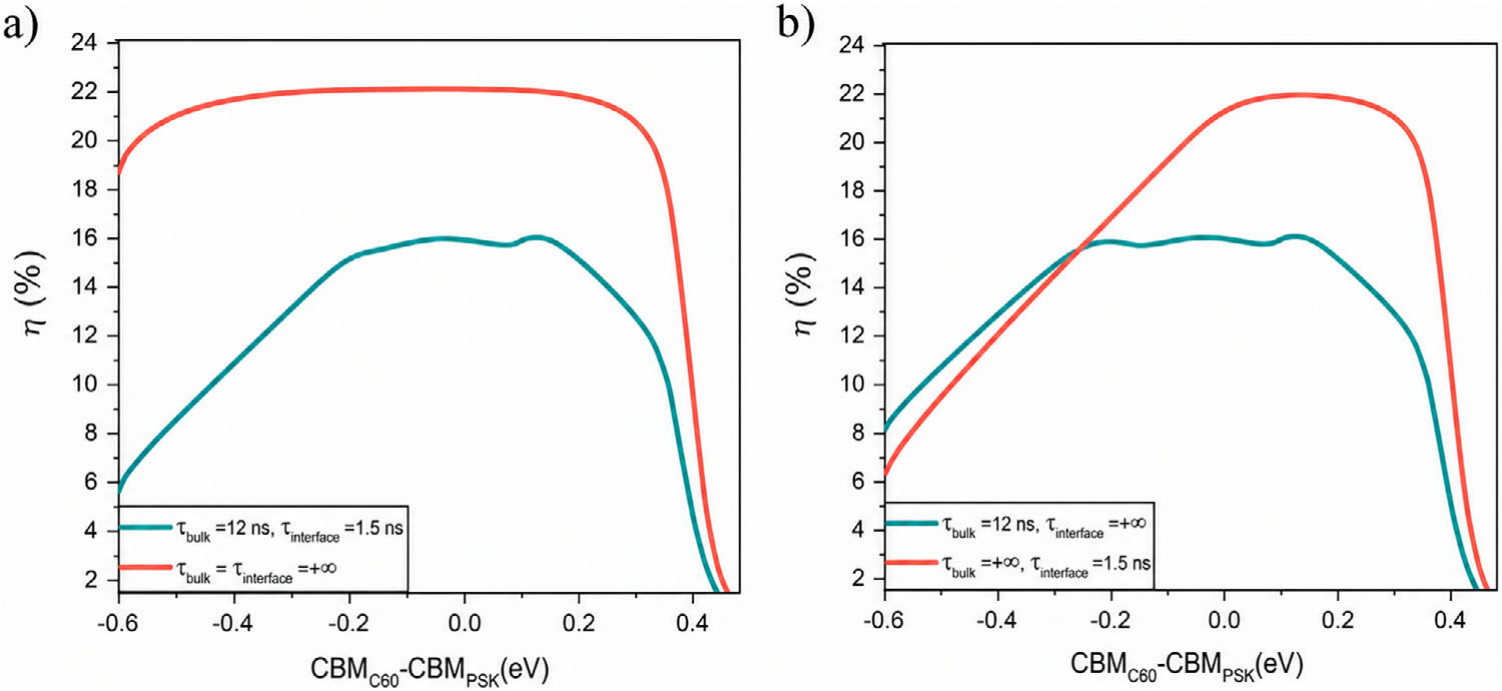
PCE of FASnI_3_ devices as a function of C_60_/perovskite layer CBO (in eV) for a p‐type doping concentration of 1 × 10^15^ cm^−3^.

To visualize the impact of non‐radiative recombination on tin perovskite solar cells, we calculated the PCE with and without the non‐radiative recombination rate determined for our devices, as illustrated in Figure 6a. The passivation of recombination channels results in a 22% improvement in PCE, highlighting the potential of TPSCs. Without addressing non‐radiative recombination, but only by resolving the ETL, we can achieve a PCE of 16%. Furthermore, we calculated the PCE by activating surface and bulk recombination individually to elucidate the contributions of surface and bulk recombination, as depicted in Figure 6b. Our analysis reveals that bulk recombination contributes dominantly to non‐radiative recombination in tin perovskite solar cells. If we resolve the interface offset, the main limitation factor of TSPCs is bulk non‐rad recombination. The doping concentration of 1 × 10^17^ itself leads only to a minor change in PCE as shown in Figure S16. Therefore, passivation and engineering of bulk defects must be considered as the primary step to achieve a PCE exceeding 20% in tin perovskite photovoltaic systems.

## 3 Conclusion

In this work, we studied the electrical, transport, and defect properties of the FASnI_3_‐based tin devices. We investigated the efficiency limiting factors of the perovskite FASnI_3_‐based solar cells. We revealed the development of their photovoltaic potential in a layer‐by‐layer completion of the ITO/PEDOT:PSS/FASnI_3_/C_60_/BCP/Ag device stack. We found that the doping concentration of a standard FASnI_3_ device using DMSO as a solvent is 1.5 × 10^17^ cm^−3^. The energy band diagram of tin perovskite devices reveals an energy offset of Δ*E*_v_ = 0.42 eV at the HTL interface and another negative band offset of Δ*E*_c_ = −0.30 eV at the ETL interface. Those offsets lead to high interfacial recombination and low charge extraction, reducing device efficiency. Consequently, better‐aligned CTLs are needed for more efficient tin perovskites.

The careful layer‐by‐layer investigation of the device stack revealed that BCP dramatically enhances electron extraction and contributes considerably to the *V_OC_* of the device under investigation. The early molecules of BCP blend with silver as a metal contact to form a charge transfer complex with an intermediate energy level of 4 eV, which is in an optimum position for electron extraction from the C_60_ to the metal contact.

Charge extraction investigations using trSPV revealed a low extraction rate in the HTL interface with a high recombination rate close to the interface compared to the bulk. In addition, the charge extraction at the FASnI_3_/PEDOT:PSS interface is driven mainly by two processes: hole extraction and electron trapping due to energy band misalignment. On the other hand, electrons show better extraction dynamics than holes, with superior performance for C_60_ over ICBA and PCBM due to the higher electron mobility regardless of the higher CBO.

Comparing tin and lead perovskites charge extraction and recombination differences shows that lead has 30 times higher electron extraction and 160 times higher hole extraction than tin perovskite. This is mainly due to the lower extraction constants as a result of energetic misalignment, which indicates the importance of enhancing the energy alignment at both interfaces. The current *p‐p‐n* structure of the tin devices leads to high interfacial charge losses because most of the charge excitations occur close to the HTL interface that has a very high band offset; however, shifting to the *n*‐*p*‐*p* structure would be more efficient due to the better extraction at the ETL interface.

We suggest designing a new charge extraction system well aligned with the tin perovskite film or trying the opposite strategy of controlling the WF of tin perovskites by polar surface additives [44] to enhance the band alignment with good CTLs such as C_60_/BCP. The methodology and findings presented in this study can be applied to identify optimal ETL and HTL candidates that enable tin perovskites to exceed the efficiency threshold of 20% and increase the commercialization possibilities of such green solar cell material.

## Conflicts of Interest

The authors declare no conflicts of interest.

## Supporting information




**Supporting File**: smll73736‐sup‐0001‐SuppMat.docx.

## Data Availability

The data that support the findings of this study are openly available in [Arxiv] at [arXiv:2309.05481], reference number [30].
