# Supplementary material for: The Role of Interfacial Energetics and Defects on The Efficiency of Tin Perovskite Solar Cells
Source: Small. 2026 May 20;22(49):e00068. doi: 10.1002/smll.202600068 (PMC13533229; doi:10.1002/smll.202600068)
Supplement: Supplementary file 1 [file SMLL-22-e00068-s001.docx]

**SUPLEMENTAL INFORMATION**

**The Role of Interfacial Energetics and Defects on The Efficiency of Tin Perovskite Solar Cells**

Mahmoud H. Aldamasy ^1, 2^‡, Artem Musiienko ^1^*‡, Marin Rusu ^1^, Davide Regaldo ^3^, Shengnan Zho ^1^, Hannes Hampel ^1^, Chiara Frasca ^1^, Zafar Iqbal ^1^, Ahmed Bedir^1,2^, Guixiang Li ^1^, Giuseppe Nasti ^4^, Meng Li ^5^, Jorge Pascual ^6^, Qiong Wang ^1^, Thomas Unold ^1^, Antonio Abate ^1,4,7^*.

^1^Helmholtz-Zentrum Berlin für Materialien und Energie GmbH. Hahn-Meitner-Platz 1, 14109 Berlin, Germany.

^2^ Egyptian Petroleum Research Institute, Nasr City, P.O. 11727, Cairo, Egypt.

^3^IPVF, Institut Photovoltaïque d'Ile-de-France, 18, Boulevard Thomas Gobert, 91120 Palaiseau, France.

^4^Department of Chemical, Materials and Production Engineering. University of Naples Federico II. Naples, pzz.le Vincenzo Tecchio 80, 80125, Naples Italy.

^5^ Key Lab for Special Functional Materials of Ministry of Education, National and Local Joint Engineering Research Center for High-efficiency Display and Lighting Technology, School of Materials Science and Engineering, and Collaborative Innovation Center of Nano Functional Materials and Applications, Henan University, Kaifeng, 475004 China.

^6^Polymat, University of the Basque Country UPV/EHU, 20018 Donostia-San Sebastian, Spain.

^7^Department of Chemistry Bielefeld University, Universitätsstraße 25, 33615 Bielefeld, Germany

‡ Those authors contributed equally

* **corresponding authors:** [antonio.abate@helmholtz-berlin.de](mailto:antonio.abate@helmholtz-berlin.de), artem.musiienko@helmholtz-berlin.de

**Experimental:**

**Materials**

Tin iodide (SnI_2_, 99.99%), tin fluoride (SnF_2_, 99%), fullerene (C_60_) bathocuproine (BCP) and indium tin oxide (ITO) were purchased from Tokyo Chemical Industry (TCI). Formamidinium iodide (FAI) was purchased from Great Cell Solar. Poly (3,4-ethylene dioxythiophene)-poly (styrene sulfonate) (PEDOT:PSS) solution (Clevios P VP Al 4083) was obtained from Heraeus Deutschland GmbH & Co. KG. Chlorobenzene (CB, anhydrous, 99.8%), dimethyl sulfoxide (DMSO, anhydrous, 99.8%), [6,6]-phenyl-C_61_-butyric acid methyl ester (PCBM), and indene-C_60_ bisadduct (ICBA) were purchased from Aldrich.

**TPSCs Fabrication**

**Tin Perovskite Cluster:**

Tin perovskites were prepared in a nitrogen atmosphere inside gloveboxes. All perovskite films and contact layers were fabricated under N₂ conditions. At HySprint Lab, Helmholtz-Zentrum Berlin for Energy and Materials, highly purified nitrogen is used for tin perovskite processing. The gloveboxes are arranged as an interconnected cluster, enabling seamless sample transfer between processing steps without exposure to oxygen. The tin-processing glovebox cluster is also equipped with several characterization tools, including a solar simulator, photoluminescence (PL), and UV–Vis spectroscopy. In addition, sample transfer between laboratories is carried out using sealed metal transfer cylinders fitted with quick-fit terminals. These cylinders are closed inside one glovebox and opened only inside another, thereby protecting the samples from exposure to air and light during transport.

**Preparation of PEDOT:PSS films:** The ITO glass substrates were cleaned through the ultrasonic bath for 30 minutes using detergent, deionized water, acetone, and ethanol sequentially. The cleaned ITO was then treated with UV-Ozone for 30 mins. Then, the ITO glass substrates were dried in an oven at 60 ℃. After that, the PEDOT:PSS layers were spin-coated on the ITO glass substrates at 4000 rpm for 40 seconds at 25 ℃ in the air, followed by annealing at 140 °C for 15 min.

**Preparation of tin perovskite precursor solutions:** 1 M FASnI_3_ perovskite precursor solutions were prepared by adding the corresponding volume from a stock 1.2 M solution of SnI_2_ in DMSO to FAI powder in a 1:1 stoichiometry. The corresponding volume from a 0.4 M SnF_2_ solution in DMSO was added to it to obtain 10% SnF_2_ concentration with respect to FASnI_3_.

**Fabrication of tin halide perovskite films:** The precursor solution was filtered by a 0.45 μm PTFE filter. Then, 100 µL filtered precursor solution was spin-coated on annealed PEDOT:PSS at 6000 rpm for 60 s using an acceleration rate of 1500 rpm/s, and 200 µL antisolvent was dropped after 55 s from the spin start. Finally, the perovskite films were annealed at 105 ℃ for 10 minutes.

**Preparation of electron transport layers:** For ICBA and PCBM, 15 mg/mL solutions in chlorobenzene were prepared and spincoated at 3000 rpm on top of the perovskite film then dried at 100℃ for 10 minutes. C_60_ was evaporated on top of the perovskite film using a vacuum evaporator. BCP and Ag were thermally evaporated afterward with a mask defining an active area of 0.16 cm^2^.

**characterization of solar cells:** J−V characteristics under 1 sun equivalent illumination were recorded using an Oriel LCS-100 class ABB solar simulator in an N_2_-filled glovebox, calibrated with a silicon reference cell from Fraunhofer ISE. The *J−V* curves were acquired with a 0.1 V s^−1^ scan rate and a voltage step of 10 mV. Devices were measured as prepared.

**Characterization:**

**Kelvin Probe** - **Photoelectron yield spectroscopy (KP**-**PYS)**

Kelvin probe (KP) and photoelectron yield spectroscopy (PYS) were combined to measure the work function (*Φ*) and ionization energy (*E*_i_) of the prepared thin films, respectively, by employing a KP Technology SKP5050-APS02 setup under N_2_ ambient. The KP system uses a 2.0 mm diameter tip with a gold alloy coating calibrated on a gold reference sample. The KP is placed in a Faraday cage, which screens the external electrical fields. The work function was determined by measuring the contact potential difference (CPD) between the Kelvin probe (reference electrode) with a known work function *Φ*_tip_, and the investigated thin film. The CPD is measured with a resolution of 3 mV. Since *Φ*_tip_ is known from reference measurements on an Au thin film, the work function of the sample, *Φ*_sample_, is calculated as:^1,2,3^

| *Φ_sample_ = Φ_tip_ + e·*CPD |  |
| --- | --- |

Where *e* is the elementary charge. The measurements of the surface photovoltage (SPV) defined as SPV = CPD_light_ − CPD_dark_ were performed using laser diodes with wavelength/energy of 405 nm/3.06 eV, 450 nm/2.75 eV, 532 nm/2.33 eV, 670 nm/1.85 eV, and 980 nm/ 1.26 eV with a light power density of ∼100 mW/cm^2^. In addition, a white light source with a spectrum of the black body at 5700K and calibrated for the light power density of ∼100 mW/cm^2^ was employed. The PYS setup uses the same Kelvin probe (static) to detect the photoemission currents as a function of incident photon energy. The light source comprises a deuterium (D_2_) lamp coupled with a grating monochromator. The range of the incident photon energy is 3.4 – 7.5 eV. The sample is illuminated via a DUV optical fiber. The ionization energy, *E*_i_, of the samples under measurement, was determined by measuring the photoelectron yield *Y*(*hν*) as a function of photon energy *hν*. *Y*(*hν*) is the number of photoemitted electrons per incident photon at a given photon energy *hν*. By extrapolating the linear part of the *Y*^1/n^(*hv*)–*hv* plot to the x-axis, the ionization energy is found according to:^1^

| *Y(hv) ∝ (hv – E_i_)^n^* |  |
| --- | --- |

The power index n = 2 for metal samples^2^ and degenerate semiconductors. For most inorganic semiconductors, n = 3.^3^. The PYS measurements were conducted with a step of 1 nm, and the photoemission threshold was determined with a resolution of 30 meV. The electron affinity (EA), i.e., conduction band minimum (CBM) referred to as the local vacuum level, is calculated as:

*EA = E_CBM_ = E_i_ – E_g_,*

where *E*_g_ is the thin film (surface) band gap.

Referred to the *E*_F_, the *E_VBM_* and *E_CBM_* are determined as:

| *E_VBM_ = Φ – E_i_,*  *E_CBM_ = (Φ – E_i_) + E_g_.* |  |
| --- | --- |

Further details on the KP and PYS methods, as well as on the combined KP-PYS setup and the applied methodology of evaluating the experimental data, can be found elsewhere^1^.

**Transient surface photovoltage (tr-SPV) measurements:**

Charge extraction in the time range of 5 ns up to 0.5 s was studied by contactless SPV measurements excited by 5 ns above bandgap laser (photon energies 1.8 and 2.6 eV). FTO probe was placed above the encapsulation glass, securing sample oxidation and at the same distance as the probed samples. trSPV data were recorded with a tunable laser in the 0.6-3 eV range using fluences of 0.07 -72 µJ. We used fluences of 0.072 µJ cm-2 for simulation, corresponding to a carrier concentration of 2×10^15^ cm^-3^ close to 1 sun operation conditions. A detailed description of the SPV setup is given in our previous study.^4^

**Simulation of charge extraction and recombination:**

**Equations S1-4:** describe the simulation model for charge separation, trapping, and recombination.

| $\frac{d\Delta p}{dt}=-K_{h}\Delta p+K_{hb}{\Delta p}_{HTM}-C_{b}\left( \left( \Delta p+p0 \right)\Delta n \right)-\frac{\Delta p}{\tau_{bulk\_h}}-s_{h}\Delta p$-$s_{ih}\Delta p$ | (Eq. S1) |
| --- | --- |
| $\frac{d\Delta n}{dt}=-K_{e}\Delta n+K_{eb}{\Delta n}_{ETM}-C_{b}\left( \left( \Delta p+p0 \right)\Delta n \right)-\frac{\Delta n}{\tau_{bulk_{e}}}+\frac{\Delta n_{t}}{\tau_{detrap}}-s_{e}\Delta n-s_{ie}\Delta n$ | (Eq. S2) |
| $\frac{d\Delta p_{HTM}}{dt}=K_{h}\Delta p-K_{hb}\Delta p_{HTM}-s_{ie}{\Delta p}_{HTM}$ | (Eq. S3) |
| $\frac{d{\Delta n}_{ETM}}{dt}=K_{e}\Delta n-K_{eb}\Delta n_{ETM}-s_{ih}{\Delta n}_{ETM}$ | (Eq. S4) |
|  |  |

Where *n* and *p* are the concentration of photo-induced electrons and holes, K_e_ and K_h_ constants correspond to electron and hole injection rates from perovskite to ETLs and HTLs. Similarly, *K*_eb_ and *K*_hb_ are reinjection rates of electron and hole to perovskite from ETL and HTL, which effectively include back tunneling/thermionic emissions and diffusion of the free carriers, as well as the drift of the free carriers due to the presence of the space charge. $C_{b}$is radiative recombination constant. $\tau_{bulk}$ do bulk defects induce SRH non-radiative recombination. $s_{e}$ and $s_{h}$characterizes non-radiative surface recombination at the perovskite surface and $s_{ie}$ and $s_{ih}$ at the HTL and ETL interface. The system of the equations cannot be solved analytically; therefore, we used the Adams backward differentiation formula (BDF) solving algorithm. We used the Levenberg-Marquardt method to fit constants according to experimental SPV results. SPV data were extrapolated logarithmically for better fitting results. The fit results are given in Figures 4b & e and summarized in Table S1 and Table S2 for tin and lead perovskite, respectively. The fixed parameters, such as doping concentration, are given in Tables S3 and S4 for tin and lead perovskite, respectively. Concentration p_0_ was found directly by Hall with AC magnetic field and other constants like relative dielectric permittivity. $\varepsilon_{ETL}$, $\varepsilon_{HTL}$, and $\varepsilon_{pero}$etc were chosen from the literature.^5,6,7^ The radiative recombination coefficient was selected from the literature.^4^

Assuming a uniform distribution of charges in perovskite ($d_{PER}\gg d_{HTM}$, $d_{PER}\gg d_{ETM}$), ETL, and HTL layers, SPV signal can be written in the form:

$SPV=\frac{L}{2}\frac{{\Delta n}_{ETM}}{\varepsilon_{ETM}\varepsilon_{0}}$+$\frac{L}{2}\frac{{\Delta p}_{HTM}}{\varepsilon_{HTM}\varepsilon_{0}}$+$\frac{L}{2}\frac{\Delta n-\Delta p-\Delta nt}{\varepsilon_{pero}\varepsilon_{0}}$(Eq. S5)

Where $L=d_{PER}/2$ -charge separation distance.

**Table S1:** Constants for tin perovskite interfaces simulation fitted according to tr-SPV data.

| Interface | *K*_h_, s^-1^ | *K*_hb_, s^-1^ | *K*_e_, s^-1^ | *K*_eb_, s^-1^ | $\boldsymbol{\tau}_{\boldsymbol{bulk}}$, ns | $\left( \boldsymbol{s}_{\boldsymbol{h}} \right)^{\boldsymbol{-}\boldsymbol{1}}$, ns | $\left( \boldsymbol{s}_{\boldsymbol{e}} \right)^{\boldsymbol{-}\boldsymbol{1}}$, ns | $\left( \boldsymbol{s}_{\boldsymbol{ih}} \right)^{\boldsymbol{-}\boldsymbol{1}}$, ns | $\left( \boldsymbol{s}_{\boldsymbol{ie}} \right)^{\boldsymbol{-}\boldsymbol{1}}$, ns |
| --- | --- | --- | --- | --- | --- | --- | --- | --- | --- |
| HTL | 7×10^4^ | 10^4^ | × | × | 12 | 10 | × | 50 | × |
| ETL | × | × | 2.6×10^6^ | 3×10^5^ | 2 | × | 400 | × | 300 |

**Table S2:** Constants for lead perovskite interfaces timulation fitted according to tr-SPV data.

| Interface | *K*_h_, s^-1^ | *K*_hb_, s^-1^ | *K*_e_, s^-1^ | *K*_eb_, s^-1^ | $\boldsymbol{\tau}_{\boldsymbol{bulk}\boldsymbol{\_}\boldsymbol{h}}$, ns | $\boldsymbol{\tau}_{\boldsymbol{bulk}\boldsymbol{\_}\boldsymbol{e}}$, ns | $\left( \boldsymbol{s}_{\boldsymbol{h}} \right)^{\boldsymbol{-}\boldsymbol{1}}$, ns | $\left( \boldsymbol{s}_{\boldsymbol{e}} \right)^{\boldsymbol{-}\boldsymbol{1}}$, ns | $\left( \boldsymbol{s}_{\boldsymbol{ih}} \right)^{\boldsymbol{-}\boldsymbol{1}}$, ns | $\left( \boldsymbol{s}_{\boldsymbol{ie}} \right)^{\boldsymbol{-}\boldsymbol{1}}$, ns |
| --- | --- | --- | --- | --- | --- | --- | --- | --- | --- | --- |
| HTL | 5×10^5^ | 10^3^ | × | × | 2000 | 74 | 6000 | × | 90000 | × |
| ETL | × | × | 9×10^6^ | 4×10^4^ | 100 | 10 | × | 10 | × | 1000 |

**Table S3:** Additional constants for tin perovskite interfaces simulation.

| Parameter | Value |
| --- | --- |
| *N (*P), cm^-3^ | 4×10^15^ |
| *C*_b_, cm^3^s^-1^ | 5×10^-10^ |
| D, nm | 300 |
| *v*_e_, cms^-1^ | 3×10^7^ |
| $\boldsymbol{\tau}_{\boldsymbol{detrap}}$, ms | >100 |
| *v_h_, cm s^-1^* | 3×10^7^ |
| *ε_per_* | 6 |
| *ε_ETL_* | 3 |
| *ε_HTL_* | 3 |

**Table S4:** Additional constants for lead perovskite interface simulation.

| Parameter | Value |
| --- | --- |
| *N (*P), cm^-3^ | 1.1×10^15^ |
| *C*_b_, cm^3^s^-1^ | 5×10^-10^ |
| D, nm | 600 |
| *v*_e_, cms^-1^ | 3×10^7^ |
| $\boldsymbol{\tau}_{\boldsymbol{detrap}}$, ms | 30 |
| *v_h_, cm s^-1^* | 3×10^7^ |
| *ε_per_* | 18 |
| *ε_ETL_* | 3 |
| *ε_HTL_* | 3 |

**Table S5:** Materials parameters used to simulate the *J*-*V* curve shown in Figure 5a.

| Parameter | Symbol | Unit | Perovskite | Perovskite  Interface -  $\mathbf{C}_{\mathbf{60}}$ | $\mathbf{C}_{\mathbf{60}}$ | $\mathbf{PEDOT}$ |
| --- | --- | --- | --- | --- | --- | --- |
| Thickness | $h$ | $\mathrm{nm}$ | $250$ | $1$ | 31 | 40 |
| Band Gap | $E_{g}$ | $\mathrm{eV}$ | 1.35 | $\chi_{PVK}+E_{g,PVK}-\chi_{C_{60}}$ | 2 | 2.2 |
| Electron Affinity | $\chi$ | $\mathrm{eV}$ | 3.48 | $\chi_{C_{60}}$ | 4.01 | 2.98 |
| Rel. Permittivity | $\epsilon$ |  | 6.5 | 6.5 | 3.6 | 3 |
| *Effective cond. band DOS* | $N_{C}$ | $\mathrm{cm}^{-3}$ | ${10}^{19}$ | ${10}^{19}$ | ${10}^{19}$ | ${10}^{19}$ |
| *Effective val. Band DOS* | $N_{V}$ | $\mathrm{cm}^{-3}$ | ${10}^{19}$ | ${10}^{19}$ | ${10}^{19}$ | ${10}^{19}$ |
| Mobility, electrons | $\mu_{n}$ | $\mathrm{cm}^{2}/Vs$ | 2.2 | 4.2 | 1.6 | 1 |
| Mobility, holes | $\mu_{p}$ | $\mathrm{cm}^{2}/Vs$ | 2.2 | 4.2 | 1.6 | 1 |
| Lifetime, electrons | $\tau_{n}$ | $\mathrm{ns}$ | 12 | 1.5 | $/$ | $/$ |
| Lifetime, holes | $\tau_{p}$ | $\mathrm{ns}$ | 12 | 1.5 | $/$ | $/$ |
| Doping (acceptor, donor) | $N_{A},N_{D}$ | $\mathrm{cm}^{-3}$ | $N_{A}={1.5\times10}^{17}$ | $0$ | $N_{D}={2\times10}^{16}$ | $N_{A}={6.7\times10}^{17}$ |
| Radiative recombination coeff | B | $\mathrm{cm}^{3}s^{-1}$ | ${9\times10}^{-11}$ | 0 | ${1.5\times10}^{-10}$ | ${1.5\times10}^{-10}$ |
| Absorption coefficient @ 500 nm | $\alpha$ | $\mathrm{cm}^{-1}$ | $1{\times10}^{5}$ | 0 | $5{\times10}^{2}$ | $5{\times10}^{3}$ |

**Figure SI 1: Types of band alignment in semiconductors.** a) Staggered gap interface, b) straddling gap interface, c) reverse staggered-gap interface and d) reverse straddling-gap interface.

**Figure SI 2**: Work function (a) and ionization energy (b) measurements of ITO on a glass substrate and PEDOT:PSS spincoated over ITO using KP and PYS, respectively.

**Figure SI 3**: The work function (a) and the ionization energy (b) of FASnI_3_ deposited on top of different substrates: Quartz, ITO, and PEDOT:PSS. This illustrates the bottom substrate’s effect on the absorber film’s energetics, band alignment, and charge extraction.

**Figure SI 4**: Work function measurement (a) and ionization energy (b) of C_60_ deposited on top of ITO.

**Figure SI 5**: The work function (a) and the ionization energy (b) of BCP deposited on top of different substrates ITO and C_60_ and even in an entire device stack. This shows the significant difference in the position of energy bands of BCP when functioning in different stacks.

**Figure SI 6**: The *J*-*V* plot of a 7.1 % PCE FASnI_3_-based device with the studied architecture.

**Figure SI 7:** The energy band diagram with respect to the Vacuum level (*E*_vac_) was plotted based on the values generated by the KP-PYS technique.

**Figure SI 8: Graphical illustration of the electron extraction mechanisms**. a) The common understanding of the BCP/Ag interface energetics. b) The energetics of the BCP/Ag interface based on our measurement in which the early evaporated nanometers blend with Ag and forms an intermediate energy level of around 4 eV that enables high capability of electron extraction.

**Figure SI 9: Illustrative diagrams of charge extraction dynamics at different excitation energies and device structures. Notably, we used two excitation energies, 688 nm (1.8 eV), which penetrates up to 210 nm, and 486 nm (2.6 eV), penetrating up to 90 nm into a 250 nm thick FASnI_3_.** a) ITO/FASnI_3_ interface. b) HTL (PEDOT:PSS or NiOx)/FASnI_3_ interface. c) Quartz/FASnI_3_/ETLs (C_60_, BCPM, and ICPA) interface. d) A full device stack illuminated from the top side.

**Figure SI 10: Illustrative explanation of the tr-SPV signal.** a) The initial ramp corresponds to the charge extraction rate. b) Signal height indicates the volume of the extracted charges, i.e., the higher the sample, the more the extracted charges will be. c) The plateau time corresponds to the recombination rate, i.e., the wider the plateau, the longer the time and the slower the recombination rate of the extracted charges.

**Figure SI 11: tr-SPV measurements of FASnI_3_ and FASnI_0.9_Br_0.1_ deposited on top of NiOx, the addition of 10% SnBr_2_ deepens the position of HOMO level which enhances band alignment.** a) Excitation near the interface, we observe a clear reduction in electron trapping when the energy bands are better aligned. b) Excitation in the bulk of the absorber film, we observe a better charge extraction.

**Figure SI 12: tr-SPV signals of FASnI_3_ deposited on top of MeO-2PACz and 2PACz compared to PEDOT:PSS using control FASnI_3_** **(Br0), FASn(I_0.9_Br_0.1_)_3_ (Br10), and FASn(I_0.8_Br_0.2_)_3_ (Br20)**. a and c) excitation using 688 nm laser pulse (1.8 eV) which penetrates deep in the film and excites electrons near from the buried interface. b and d) Excitation using 486 nm laser pulse (2.6 eV) exciting electrons in the bulk of the perovskite film.

**Figure SI 13: Comparison between tr-SPV signals of different device stacks.** a) tr-SPV signals of FASnI3 with HTL and ETL, we observe a big jump in charge extraction after the insertion of both CTLs and much bigger after the insertion of BCP. b) The insertion of NiOx does not enhance the charge extraction as compard to a due to the low efficiency of NiOx in extracting holes leading to higher recombination and lower extraction as indicated by the lower tr-SPV signals.

**Supplementary note 1:** Electron extraction dynamics in sandwiched FASnI_3_ between NiOx and different ETLs.

To deeply understand electron extraction dynamics in tin perovskites, we prepared FASnI_3_ films sandwiched between ETLs and HTLs together. Such stacks enable us to study the mutual effect between ETLs and HTLs and, moreover, they are very close to the real operation conditions of the full device stack where electrons and holes are extracted together at the same time. **In Figure SI10**, we prepared the same device stack one based on PEDOT:PSS as HTL and the other is based on NiO_x_. In the case of PEDOT:PSS, we followed the evolution of the tr-SPV signal by measuring the device stack layer by layer, however in the NiO_x_ case, we measured different ETLs to check their effect on charge extraction when the HTL layer is inserted. By comparing the signal amplitude of the two stacks ITO/PEDOT:PSS/FASnI_3_/C_60_ and ITO/NiO_X_/FASnI_3_/C_60_, we find that, in the PEDOT:PSS case, we extract more than double the charges extracted in the case of NiO_x_. Furthermore, we observe much slower recombination dynamics in the case of PEDOT:PSS (10 ms) compared to NiOx (28 μs). Conclusively, the efficiency of the HTL in extraction holes affects electron extraction significantly, as it decreases holes density in the absorber material and in turn the recombination probability. **Figure SI 10a** shows a big jump in electron extraction after BCP was inserted, which agrees with the KP SPV signal showed in **Figure 1e**, both measurements confirm the vital role of BCP in electron extraction as we illustrated earlier. **Figure SI 10b** shows the electron extraction with NiOx and different ETLs, it has almost the same trend without HTL as shown in **Figures 3c-d**. However, C_60_ shows even faster recombination dynamics leading to a positive pump which has a maximum amplitude of around 6 mV at 5 μs. This could be attributed to the electrons trapping at the FASnI_3_/NiOx layer, which retards the charges recombination across the FASnI_3_ film at the beginning, then leaves holes as the majority of charges in the perovskite film, which is recorded in the measurement as a positive signal then electrons are slowly released and recombine with holes. In addition, this positive signal appears only with C_60_ compared to other ETLs due to its higher charge mobility. As a result, C_60_ releases electrons faster than PCBM and ICBA, i.e., extracted electrons in C_60_ are released and recombined before the trapped electrons in the NiOx are released, as illustrated in **Figure SI 11**.

**Figure SI 14: Graphical illustrations of the charge carrier dynamics of the device stack NiOx/FASnI_3_/C_60_**. a) Electrons are trapped at the NiOx/FASnI_3_ interface upon excitation. b) Holes are traveling to recombine in the bulk of the perovskite absorber. c) Holes are the majority in the bulk of the absorber because electrons are trapped in the NiOx, leading to the appearance of the positive SPV signal. d) Finally, trapped electrons are released and recombine with holes.

**Simulation of tin perovskite solar cell operation**

We have employed Atlas, a 2D drift-diffusion simulator provided by Silvaco®, to develop a physics-based model of the functioning of the tin perovskite solar cell stack under investigation. We have modeled the perovskite, C_60_, and PEDOT:PSS as crystalline semiconductors with their thickness, energy band gap, and affinity following the findings above by KP and PYS. We have set the perovskite p-type doping to ${1.5\times10}^{17}cm^{-3}$, as demonstrated by PL, TRPL, and Hall effect measurements in this work. We have also added bulk non-radiative recombination in the perovskite, with a lifetime of 12 ns. We have set the effective density of states of the materials at 10^19^ cm^-3^. We have set the relative permittivity values for the perovskite^7^, to for C_60_^5^, and to for PEDOT:PSS^6^. The C_60_ and PEDOT:PSS absorption coefficient values were taken as average values from the literature.

A large valence band offset was measured between the perovskite and PEDOT:PSS (about 0.47 eV). In the model, to allow photogenerated holes in the perovskite to be injected in PEDOT:PSS via thermionic emission over the barrier, we had to increase the valence band maximum (VBM) of PEDOT:PSS by 0.12 eV with respect to our experimental data. This is carried out to mimic possible defect-assisted tunneling across the barrier. We also note that the VBM edge of PEDOT:PSS, detected on its bare surface, may shift upon perovskite film deposition. We have set the p-type doping density of PEDOT:PSS to reproduce the VBM-E_F_ energy distance detected by KP and PYS.

To fit the experimental *J*-*V* curve in **Figure 5a** (black line), we have varied the carrier mobility of the perovskite and PEDOT:PSS, as well as the doping value of C_60_, the perovskite radiative recombination coefficient, and the perovskite absorption coefficient. We also inserted Schottky contacts at the C_60_ and PEDOT:PSS side, with work function (WF) values of 3.95 and 4.74 eV, respectively. These values correspond to the measured WF of ITO and Ag:BCP and provide a good fitting of the FF of the *J*-*V* curve.

Due to the considerable conduction band offset (CBO) between the perovskite and C_60_ (about -0.53 eV), we expect a high non-radiative recombination rate at this interface. Therefore, we added a fictitious layer between these two materials with Shockley-Read-Hall non-radiative recombination. This 1-nm-thick layer shares the conduction band minimum (CBM) of C_60_, and the valence band maximum of the perovskite, thus allowing for the recombination between photogenerated electrons injected in C_60_ with photogenerated holes in the perovskite. This layer was necessary to reduce the open circuit voltage of our simulated perovskite solar cell from about 0.9 V (PCE=12.7%) to values close to the experimental data.

We report all the material parameters employed to produce the blue *J*-*V* curve in **Figure 5a** in **Table S5**, together with the experimental curve (black line). **Figure 5a** also contains the *J*-*V* curve obtained by the same parameter choice in the n-i-p configuration, where the material stack is illuminated from the C_60_ side (orange curve).

**Figure SI 15:** Band diagram at the equilibrium of the solar cell as a function of perovskite doping (3 * 10^15^ cm^-3^ (a), 3 * 10^16^ cm^-3^ (b), 3 * 10^17^ cm^-3^ (c)). The rest of the parameters employed are defined in **Table S5**.

**Figure SI 16:** PCE of FASnI_3_ devices as afunction of the conduction band offset (CBO) at two different doping concentrations.

**Figure SI 17:** SEM top view of FASnI_3_ films confirming the compactness of the prepared devices. a) FASnI_3_/Quartz, b) FASnI_3_/PEDOT, and c)FASnI_3_/NiOx. Scale bar is equivalent to 300 nm.

**Figure SI 18:** TRPL measurements of FASnI_3_/glass films.

**References:**

(1)	Rusu, M.; Kodalle, T.; Choubrac, L.; Barreau, N.; Kaufmann, C. A.; Schlatmann, R.; Unold, T. Electronic Structure of the CdS/Cu(In,Ga)Se_2_ Interface of KF-and RbF-Treated Samples by Kelvin Probe and Photoelectron Yield Spectroscopy. *ACS Appl. Mater. Interfaces* **2021**, *13*, 7755.

(2)	Fowler, R. H. The Analysis of Photoelectric Sensitivity Curves for Clean Metals at Various Temperatures. *Phys. Rev.* **1931**, *38* (1), 45.

(3)	Ballantyne, J. M. Effect of Phonon Energy Loss on Photoemissive Yield near Threshold. *Phys. Rev. B* **1972**, *6* (4), 1436. https://doi.org/10.1103/PhysRevB.6.1436.

(4)	Levine, I.; Al-Ashouri, A.; Musiienko, A.; Hempel, H.; Magomedov, A.; Drevilkauskaite, A.; Getautis, V.; Menzel, D.; Hinrichs, K.; Unold, T.; Albrecht, S.; Dittrich, T. Charge Transfer Rates and Electron Trapping at Buried Interfaces of Perovskite Solar Cells. *Joule* **2021**, *5* (11), 2915–2933.

(5)	Sami, S.; Haase, P. A. B.; Alessandri, R.; Broer, R.; Havenith, R. W. A. Can the Dielectric Constant of Fullerene Derivatives Be Enhanced by Side-Chain Manipulation? A Predictive First-Principles Computational Study. *J. Phys. Chem. A* **2018**, *122* (15), 3919–3926.

(6)	Zhao, Y.; Zhao, Y.; Zhao, Y.; Lin, Y.; Lin, Y.; Zeng, Z.; Su, C.; Jiang, C.; Jiang, C. Determination of the Dynamic Dielectric Function of PEDOT:PSS from the Visible to the near-Infrared Region. *Opt. Mater. Express, Vol. 11, Issue 9, pp. 3049-3055* **2021**, *11* (9), 3049–3055.

(7)	Pachori, S.; Agarwal, R.; Prakash, B.; Kumari, S.; Verma, A. S. Fundamental Physical Properties of Nontoxic Tin-Based Formamidinium FASnX3 (X = I, Br, Cl) Hybrid Halide Perovskites: Future Opportunities in Photovoltaic Applications. *Energy Technol.* **2022**, *10* (2), 2100709.
